# Decision tool of medical endoscope maintenance service in Chinese hospitals: a conjoint analysis

**DOI:** 10.1186/s12913-023-10458-y

**Published:** 2023-12-15

**Authors:** Jun Zheng, Jingming Wei, Ying Xie, Siyao Chen, Jun Li, Ligang Lou, Jing Sun, Jingyi Feng

**Affiliations:** 1 Key Laboratory of Clinical Evaluation Technology for Medical Device of Zhejiang Province, The First Affiliated Hospital, Zhejiang University School of Medicine, Hangzhou, Zhejiang China; 2 Peking University Institute of Mental Health, Beijing, 100191 China; 3 School of Management, Cranfield University, College Road, Cranfield, Bedford, MK43 0AL UK; 4 Department of Clinical Engineering and Material Supplies, The First Affiliated Hospital, Zhejiang University School of Medicine, Hangzhou, Zhejiang China

**Keywords:** Medical endoscope, Maintenance service demands, Decision-making, Conjoint analysis

## Abstract

**Background:**

Medical devices are instruments, apparatus, appliances, software, implants, reagents, materials or other articles that are intended for use in the treatment or diagnosis of disease or injury in humans. Concerning medical endoscope devices, which enable doctors to observe and manipulate the area under examination through a puncture hole in the body cavity or organ, hospitals predominantly consider the quality and cost of maintenance services when making their selection. The effective and efficient provision of maintenance services plays a crucial role in ensuring cost-effective and high-quality management of medical devices. In this study, we have developed an innovative decision tool that analyzed key factors impacting the choice of medical devices’ maintenance service. This tool assists hospitals in evaluating and selecting appropriate maintenance services for medical device, specifically endoscopy devices. Moreover, it also serves as a valuable resource for manufacturers and suppliers to enhance their after-sales service offerings.

**Methods:**

A cross-sectional survey was undertaken in 50 Chinese hospitals, including primary and tertiary hospitals. Moreover, 56 medical staff and 65 medical engineers were recruited from 50 Chinese hospitals to participate the survey. A comprehensive set of factors were defined and investigated. Conjoint analysis and orthogonal design were used for survey design and statistical analysis.

**Results:**

Factors importance and utility values of decision-making factors were analyzed at the aggregate, occupation, and medical institution levels. (1) At the aggregate level, the most critical factor is “maintenance response” and the least important one is “maintenance efficiency”. (2) At the occupation level, medical staff paid more attention to “maintenance response” while medical engineers paid more attention to “maintenance quality”. (3) At the medical institution level, Primary hospitals paid more attention to “maintenance price”, while tertiary hospitals paid more attention to “maintenance quality”.

**Conclusions:**

In general, this study provides a more scientific decision-making tool to both hospitals in choosing maintenance service for medical device such as endoscopy, and it also helps manufacturers and suppliers improve the after-sales service.

**Supplementary Information:**

The online version contains supplementary material available at 10.1186/s12913-023-10458-y.

## Background

Medical devices or medical equipment are instruments, apparatus, appliances, software, implants, reagents, materials, or other articles that are intended for use in the diagnosis, prevention, monitoring, treatment, or alleviation of disease or injury in humans [1]. Maintenance service of medical device is crucial to ensure that a device operates in accordance with manufacturer specifications and aids diagnosis and treatment of medical conditions [2, 3]. The actual costs associated with medical devices include purchase, maintenance, and reprocessing costs [4, 5]. The maintenance of medical devices is also important for reducing the overall dispatch costs, ensuring timely patient treatment, and reducing mortality and risks during patient care [6, 7].

Medical endoscope is a kind of medical device that allows doctors to observe and manipulate the area being examined through a puncture hole in the body cavity or organ [8]. As a medical device, the endoscopes must go through risk assessment which is an ongoing responsibility and must be managed as a top priority by manufacturers, suppliers (agents selling endoscopes), and hospitals [9]. The maintenance service provided by manufacturers or suppliers is regarded as essential service and support for endoscopes, such as Repairing, Pre-Maintaining, and Quality-Control. Good maintenance of medical devices must be implemented by hospitals to minimize device breakdowns or failures [10], and to make sure they are accessible and reliable when needed [11]. This is even more critical for developing countries, such as China, which have much lower health expenditure per capita compared with developed countries [12].

The State Food and Drug Administration has introduced some national and industrial standards to audit the quality of medical endoscope products [13, 14]. Arab-Zozani et al. (2021) [10] also developed assessment checklists for medical device maintenance management, from the aspects of resources, service, education, quality control, inspection, and preventative maintenance information management. However, the checklist was proposed using the Iranian experience, and it may not apply to other countries. Nor has the checklist been tested in a real hospital context, hence the validity, reliability, and feasibility of the checklist require further examination. Furthermore, the factors included in the checklist are rather generic, and they do not specify factors associated with inspection and maintenance service, such as response time, cost of inspection or repair, etc.

The primary goal of this research is to evaluate the maintenance service quality of medical endoscopes, Several industry associations in China have explored various assessment methods and conducted multiple demand surveys on the maintenance service of medical endoscopes. For example, Shanghai medical device quality control center conducted service evaluation and demand analysis of medical endoscopes as early as 2011 [15–17]. Assessment of service quality requires information concerning service to produce aggregate assessment scores or metrics. The ranking method proves to be a popular assessment method used to evaluate the quality of service to medical devices [18]. Information relevant to service attributes is usually collected from systematic literature reviews or surveys [10].

We need to develop a method that considers a comprehensive list of the attributes associated with medical endoscopes and determines the most effective or essential combination of attributes [19–21].

This research collected information concerning the maintenance service provided to medical endoscopes, through an experimental design and a conjoint survey. The survey was conducted with a representative sample of medical staff and medical engineers across a subset of Chinese hospitals. The questionnaire was distributed via emails and social media platforms. Using conjoint analysis, we analyzed specific needs for endoscope maintenance service by comparing rankings between medical staff and medical engineers and between tertiary hospitals and primary hospitals. In the hospital context, we further explored the factors influencing the decision-making of purchasers, end-users, and maintenance managers when choosing endoscope products and services. The research will facilitate decision-making at hospitals in choosing the suitable endoscope device and the associated maintenance service; it also offers a framework to set up standards for maintenance service. The ultimate objective of this research is to advance the field of industrial design for medical endoscope products, with a focus on enhancing maintenance service level and effectiveness. Additionally, it seeks to drive continuous product improvement within the after-sales service system related to these products [22, 23].

## Methods

### Respondents

To reduce the impacts of selection bias, the sampling method used in choosing respondents was random, and the professionals who met the criteria had an equal probability of being enrolled through a non-probabilistic, convenience sampling method.

We conducted the conjoint survey across 50 hospitals in different provinces in China, including both primary and tertiary hospitals.

The selection criteria of participants are: (1) Participants in this study were professionals who worked in hospitals that have utilized medical endoscopes and procured maintenance services within the past five years. (2) The participants in this study included medical staff (including doctors and nurses) as well as medical engineers who had more than three years of experience in using or managing endoscopes. This study constitutes a part of one major research project sponsored by the Ministry of Science and Technology of China, running from 2017 to 19. Over the course of three years, participant recruitment for the project was successfully accomplished through various channels, including sending targeted emails and messages via social media platforms like WeChat. Ultimately, 50 Chinese hospitals entered into agreements to take part in both the primary research project and this study.

### Experimental method

Experimental design refers to the process of generating specific combinations of factors and levels evaluated by respondents. In this study, conjoint analysis and orthogonal design were used for experimental design and statistical analysis. Conjoint analysis is a survey-based statistical technique used to help determine how people evaluate different factors of products or services, such as functions and features [24]. The conjoint analysis presents choice alternatives between products or services defined by a combination of factors; it can also be used to determine each factor’s relative importance and which levels of each factor are most preferred. In conjoint analysis, each profile describes a complete product or service, and it is defined by a different combination of factor levels for all factors of interest. The full-profile approach is used in conjoint analysis, where respondents score, rank, or order a set of profiles. If the number of combinations of factor levels is too large, a fractional factorial design is introduced to deal with the problem. A fractional factorial design, also called orthogonal design, selects a fraction of all possible combinations of factor levels to capture the main effects for each factor level. The Orthogonal design is typically a starting point of a conjoint analysis [25, 26]. The rest of the combinations that are not used in the orthogonal design are called holdout profiles.

In an orthogonal design, we assume there are K factors, and each factor has n levels, i.e., t1, t2, …, tn. If this design meets two conditions: ① different levels of each factor appear the same number of times in the test (equilibrium); ② different combinations of factor levels for any two factors appear the same number of times in the test (orthogonality), this design is called orthogonal design. The orthogonal design is used to generate an orthogonal array, which can make the distribution of test points very uniform and reduce the number of tests. In this study, an orthogonal array is used to generate factor-level combinations of profiles, also called cards, which are rated by the respondents (also called subjects).

A random sample of subjects (respondents) from the target population is selected to evaluate the set of profiles or cards. The subjects assign a preference score to each profile based on intuitive experience. The reference score can be a Likert scale or a number between 1 and 100. Alternatively, subjects can assign a rank to each profile using a number from 1 to the total number of profiles.

The survey data results are analyzed in a utility score, called part-worth, which provides a quantitative measure of preference for each factor level. Each factor has multiple levels; we are interested in each factor’s preference value or relative importance. The calculation of factor importance value is presented in a multivariate framework [27, 28]:

1 $$Z(x) = {e_{ij}} + \sum\nolimits_{i = 1}^k {\sum\nolimits_{j = 1}^n {Uij * Xij} }$$

Where Z(x) is the overall utility for a card (profile), rated by the subjects; U_ij_ represents the part-worth utilities for factor level j of factor i; X_ij_ represents the level of a factor; it is a categorical variable, measuring weather factor i at level j is absent (= 0) or present (= 1) in this card (profile). K is the number of factors; n is the number of levels in each factor. U_ij_ is the value of interest, and it is estimated using the Ordinal Least Square method using the linear regression model. And e_ij_ is the stochastic error term.

Once utility score U_ij_ is obtained, the range of the utility score for a factor i is calculated as the difference between the maximum and the minimum party worth utility:

2 $$Range\,of\,Utility\,Score\,of\,factor\,i\, = \,max\,\left( {Uij} \right) - min\,\left( {Uij} \right)$$

The importance value of factor i is expressed as:

3 $$\frac{{Range\,of\,Utility\,Score\,of\,factor\,i}}{{\sum\nolimits_{i = 1}^K {Range\,of\,Utility\,Score\,of\,factor\,i} }}$$

The factor importance value ranges between 0 and 1. The greater the value, the more important of the factor in the evaluation system of endoscope maintenance service.

Conjoint analysis is a valuable market research technique for understanding consumer preferences and making informed product or service design decisions. However, like any research method, it comes with its own set of advantages and disadvantages. Advantages of conjoint analysis include: (1) Simulating real world decision making scenarios for respondents and providing realistic insights into how consumers prioritize factors. (2) Quantifying the importance of each factor and its level by examining how changes in these factors affect choice probabilities. This information helps business focus on enhancing the most important factors for consumers. (3) Identifying the trade-offs based on consumers’ preferences on different factors, which can guide product development and marketing strategies; The joint analysis method also has some limitations: since it only considers factors and levels included in the conjoint design, potentially missing out on other important factors that influence consumer choices. The quantified scores are based on the subjective preferences of respondents, which could be biased and sensitive to the definition of samples. Hence, this research enhanced the conjoint analysis approach through the integration of several research methods, including the Delphi method and orthogonal design [29–31].

In this study, the combinations of different factors and related levels of medical endoscopes were created based on the results from the Delphi method [32]. The set of profiles (cards) was created through orthogonal experimental design, which required the subjects (respondents) to assign preference to each combination intuitively based on experience; then, the importance of each factor and the effect of factor level are calculated using Eqs. (1)-(3).

### Research process

#### Cross-sectional survey

Since little was known about preferences for different factors of endoscope maintenance, a cross-sectional survey was adopted to obtain a snapshot of the participant’s views on the endoscope maintenance service. A cross-sectional survey was conducted in 2019 to assess the participants’ preferences for endoscopes maintenance service. The advantages of cross-sectional surveys are fast and cost-effective. However, this is a one-off measurement over a short period; it is challenging to derive causal relationships among factors based on the cross-sectional survey results.

#### Conjoint analysis and orthogonal experiment design

To inform questionnaire design, we selected combinations of different factors and related levels of the medical endoscope from the factors reported in which used two rounds of the Delphi method to assess the service level of endoscopes [32]. A set of factors were identified from the Delphi method for medical endoscope maintenance service, including maintenance quality, maintenance price, maintenance response, maintenance efficiency, and service provider. These factors are defined as following.

1. Maintenance quality encompasses the normal operation, reliability, safety, stability, and service life of repaired medical endoscopes. In this study, the Mean Time Between Failure (MTBF) is used to assess the maintenance quality for the same fault phenomenon.
2. Maintenance price refers to the cost associated with a single repair of a medical endoscope, including labor costs, parts costs, transportation costs, and other related expenses.
3. Maintenance response pertains to the duration it takes for service provider to reach the service site following the receipt of a fault report from the user of a medical endoscope.
4. Maintenance efficiency denotes a medical endoscope service provider’s capability to complete repair tasks within a specified timeframe. In this study, it specifically refers to the duration required for the maintenance service, encompassing the entire repair process from initiation to completion.
5. Service provider refers to a company, organization, or individual that offers medical endoscope repair services. In this study, it is categorized into two types: original equipment manufacturers (OEMs) and third-party service providers.

Each factor contains multiple levels, and Table 1 illustrates the meaning of each factor and its corresponding levels. For example, there were three levels of maintenance quality, defined as “the same fault happened within 6 months”, “the same fault happened between 6 months and 12 months”, or “the same fault happened after 12 months. Maintenance prices varied with the hard endoscope and soft endoscope, as defined at the three levels. Similarly, maintenance response rate and efficiency were also measured at three levels. Service provider was classified as the service provided by the original manufacturer or by the third-party service agents.

**Table 1:** Endoscope maintenance service factors and levels

Factor	Level
**Maintenance quality**	1. Same fault ≤ 6 months
	2. 6 months < Same fault ≤ 12 months
	3. Same fault > 12 months
**Maintenance price**	1. Rigid endoscope ≤ 5000 yuan, Flexible endoscope ≤ 10,000 yuan
	2. Rigid endoscope ≤ 10,000 yuan, Flexible endoscope ≤ 30,000 yuan
	3. Rigid endoscope ≤ 20,000 yuan, Flexible endoscope ≤ 500,000 yuan
**Maintenance response**	1. Response ≤ 1 day
	2. 1 day < Response ≤ 3 days
	3. 3 days < Response ≤ 7 days
**Maintenance efficiency**	1. Maintenance ≤ 10 days
	2. 10 days < maintenance ≤ 20 days
	3. 20 days < maintenance ≤ 30 days
**Service provider**	1. Service provided by the original manufacturers
	2. Third-party service providers

The questionnaire contains three sections (see Table S1 in the Supplementary Information): (a) demographic information of the respondents, including employer information, occupation, number of years of working, Etc.; (b) an explanation of the maintenance service factors and levels, as well as the type of method used to assign preference scores; and (c) the main body of the questionnaire, presenting the combinations (also named as cards or profiles) of the factor levels. Each respondent was asked to answer the question “how likely would you choose the above service?” using the ten-level Likert scale (score 1–10).

**Fig. 1:**
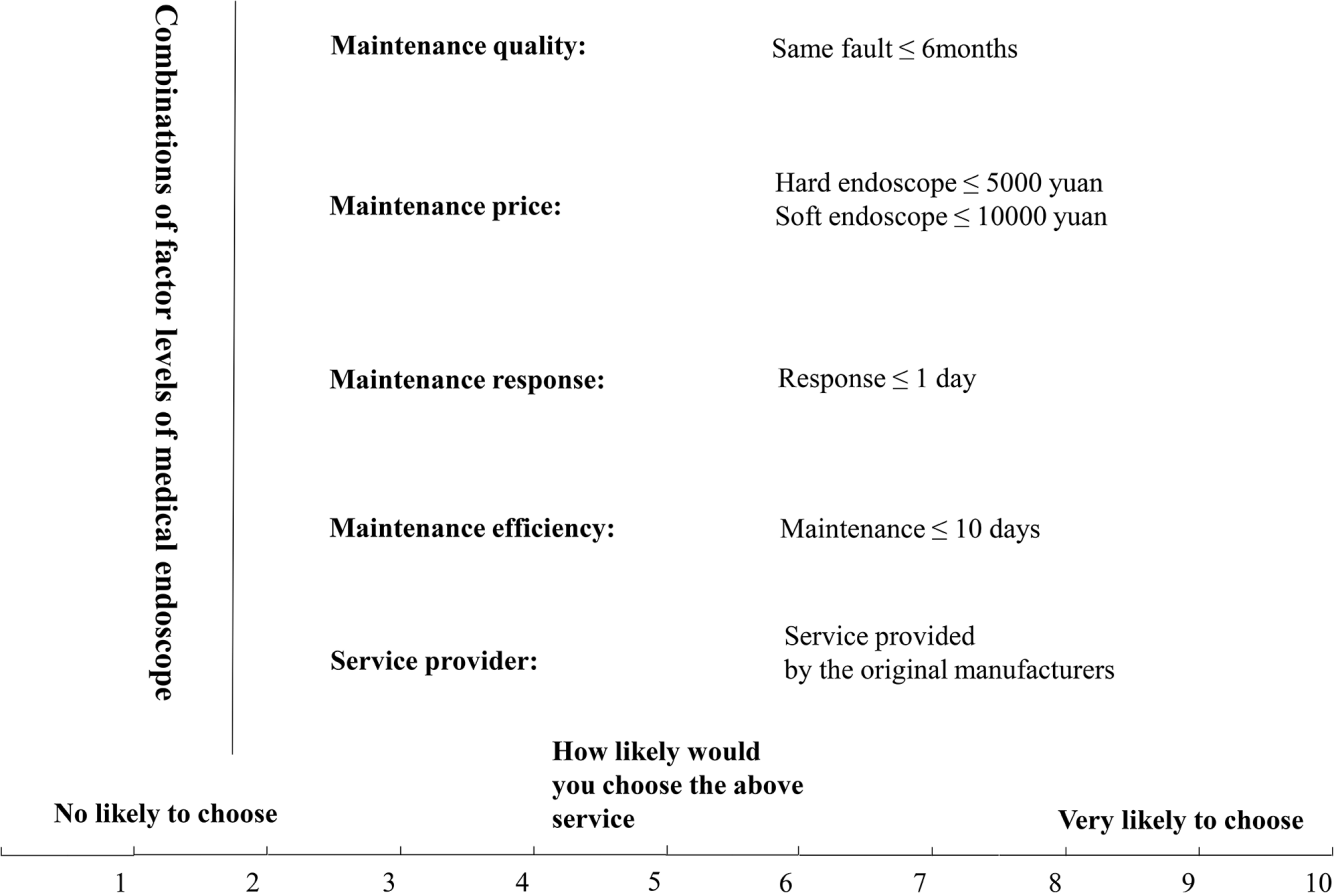
A profile (card) of endoscope maintenance service

The full-profile approach of Conjoint analysis generates 162 (3 × 3 × 3 × 3 × 2) profiles resulting from all possible combinations of the factor levels. The total number of 162 became too big for respondents (subjects) to rank or score in a meaningful way. So, the orthogonal design was used to reduce the number of combinations and retain the main effects of combinations that reflect the service factors of medical endoscopes. The orthogonal experimental design was carried out in SPSS software, and a reduced set of 16 profiles (cards) were generated. The 16 cards represented different combinations of factor levels of the medical endoscope, and the sample card is shown in Fig. 1. As we could see in Fig. 1, the number of 16 profiles was small enough to include in a survey but big enough to assess the relative importance of each factor [33].

## Results

A total of 125 questionnaires were distributed, with 121 returned, all of which were deemed valid, resulting in an effective response rate of 96.8%. As shown in Table 2, the 121 participants were comprised of 56 medical staff and 65 medical engineers. Within this group, 27 individuals represented primary hospitals, while 94 came from tertiary hospitals. All participants had received education at the undergraduate level or higher, with an average of 16.8 years of work experience in the relevant field. Among them, medical staff had an average of 17.3 years of experience, while medical engineers had an average of 16.1 years.

**Table 2:** Demographic analysis of participants

Statistical project	Gender (count)		Education (count)		Occupation (count)		Hospital employer (count)		Working experiences(year)	
Statistical results	Male:	79	Bachelor’s degree or higher:	121	Medical staff:	56	Primary hospital:	27	Medical staff:	17.3
	Female:	42	Other:	0	Medical engineer:	65	Tertiary hospital:	94	Medical engineer:	16.1

Table 3 shows the utility values and factor importance scores of endoscope maintenance service rated by different participants, i.e., medical staff, medical engineers, and the whole medical staff and medical engineers’ population. To verify the validity of the conjoint model, this study provided goodness-of-fit measures to determine if the hospitals behave according to their preferences. The internal validity of the conjoint analysis was worked out based on the correlations of the average rating score from the hold-out responses and the predicted levels of utility. In this study, the Pearson correlation coefficient was 0.875 (*p* > 0.001), and Kendall’s tau was 0.662 (*p* > 0.001), indicating the conjoint model has a good fitting.

**Table 3:** Utility value and factor importance rated by participants with different occupations

Factor	Level	Medical staff			Medical engineers		Population	
		Utility value	Factor Importance (%)		Utility value	Factor Importance (%)	Utility value	Factor Importance (%)
**Service provider**	1. by the original manufacturer	0.450	16.546		0.313	18.923	0.373	17.873
	2. By third party service providers	-0.450			-0.313		-0.373	
**Maintenance quality**	1. Same fault ≤ 6 months	-2.811	22.612		-2.601	21.811	-2.694	22.165
	2. 6 months < same fault ≤ 12 months	-3.847			-3.565		-3.690	
	3. Same fault > 12 months	-3.106			-2.894		-2.988	
**Maintenance price**	1. Rigid endoscope ≤ 5000 yuan, Flexible endoscope ≤ 10,000 yuan	1.774	20.396		1.011	21.408	1.348	20.961
	2. Rigid endoscope ≤ 10,000 yuan, Flexible endoscope ≤ 30,000 yuan	2.469			1.416		1.881	
	3. Rigid endoscope ≤ 20,000 yuan, Flexible endoscope ≤ 50,000 yuan	2.087			1.215		1.600	
**Maintenance response**	1. Response ≤ 1 day	1.547	24.328		1.519	23.141	1.531	23.665
	2. 1 day < response ≤ 3 days	2.392			2.280		2.329	
	3. 3 days < Response ≤ 7 days	2.533			2.284		2.394	
**Maintenance efficiency**	1. Maintenance time ≤ 10 days	1.226	16.118		1.071	14.717	1.140	15.336
	2. 10 days < maintenance time ≤ 20 days	1.762			1.534		1.634	
	3. 20 days < maintenance time ≤ 30 days	1.606			1.388		1.484	

Table 4 shows the utility values and factor importance scores of endoscope maintenance service rated by different hospitals, i.e., primary hospitals, tertiary hospitals, and the total hospitals.

**Table 4:** Utility value and Factor importance rated by participants from different medical institutions

Factor	Level	Primary hospital		Tertiary hospital		Population	
		Utility value	Factor Importance(%)	Utility value	Factor Importance(%)	Utility value	Factor Importance(%)
**Service provider**	1. by the original manufacturer	0.350	16.378	0.380	18.307	0.373	17.873
	2. by third party service providers	-0.350		-0.380		-0.373	
**Maintenance quality**	1. Same fault ≤ 6 months	-2.639	20.399	-2.710	22.678	-2.694	22.165
	2. 6 months < Same fault ≤ 12 months	-3.579		-3.722		-3.690	
	3. Same fault > 12 months	-2.819		-3.036		-2.988	
**Maintenance price**	1. Rigid endoscope ≤ 5000 yuan, Flexible endoscope ≤ 10,000 yuan	1.620	21.479	1.269	20.811	1.348	20.961
	2. Rigid endoscope ≤ 10,000 yuan, Flexible endoscope ≤ 30,000 yuan	2.181		1.794		1.881	
	3. Rigid endoscope ≤ 20,000 yuan, Flexible endoscope ≤ 50,000 yuan	1.681		1.577		1.600	
**Maintenance response**	1. Response ≤ 1 day	1.250	26.946	1.613	22.713	1.531	23.665
	2. 1 day < response ≤ 3 days	1.958		2.437		2.329	
	3. 3 days < Response ≤ 7 days	2.125		2.472		2.394	
**Maintenance efficiency**	1. Maintenance time ≤ 10 days	0.880	14.799	1.215	15.492	1.140	15.336
	2. 10 days < maintenance time ≤ 20 days	1.292		1.734		1.634	
	3. 20 days < maintenance time ≤ 30 days	1.236		1.556		1.484	

### Preferences of maintenance service of medical endoscope

According to the results of the conjoint analysis, the primary factor influencing medical staff and engineers’ satisfaction with maintenance service is the “maintenance response” (23.665%), followed by “maintenance quality” (22.165%), “maintenance price” (20.961%), “service providers” (17.873%), and “maintenance efficiency” (15.336%), as shown in Table 3.

In Table 3, the utility values of the factor level reflected the respondents’ preference for the service selection. The greater the absolute value of the utility, the stronger the preference. The total population’s preferences on endoscope maintenance service had the following features: for the factor of “service provider”, there was a big difference between the two levels. Compared with the other two factor levels, the medical staff and engineers were more willing to accept the moderate level of maintenance quality, as the absolute value of this factor level is the highest (3.690). The factor “maintenance response” had three levels of positive utility values, of which the utility value of “3 days < maintenance response ≤ 7 days” received the highest score of 2.394. This result means that moderate maintenance response and receiving maintenance response within 7 days was most describable. The factor of “maintenance efficiency” also had three levels of positive utility values, of which the factor level “10 days < maintenance time ≤ 20 days” received the highest utility value of 1.634, showing that this level of efficiency was most desirable for medical staff and engineers.

### Analysis of preferences on service profiles

The results showed that medical staff and engineers tend to choose the service provided by the original manufacturer, with quick response time, short maintenance time, and low price. Compared with the other four factors, a moderate level of maintenance quality (6 months <the same fault ≤ 12 months) was generally acceptable to the medical staff and engineers. Medical endoscopes are operated on the human body with a high frequency of use, and the operating environment is complex. Therefore, there is usually a high failure rate, with an average maintenance frequency of once per 12 months. The least favorable service profile was characterized by the *third-party service providers* and poor *maintenance quality* (“the same fault ≤ 6 months”), which was consistent with the actual expectations on endoscope maintenance service, i.e., there was strong resistance to the third-party maintenance service and poor maintenance service.

### Analysis of preferences by respondents with different occupations

As shown in Table 3, the results showed no significant difference in factor importance or factor utility values between medical staff and medical device engineers. The ranking of factor importance was consistent between the two groups, showing that the selection preferences were identical in terms of *service provider*, *maintenance quality*, *maintenance price*, *maintenance response*, and *maintenance efficiency*. The utility values of factor levels varied between medical staff and engineers.

When choosing service provider, both groups preferred the service provided by the original manufacturer. However, medical engineers felt a slight difference between the maintenance service provided by the original manufacturer and the service provided by the third-party provider, while medical staff thought that the difference was significant.

### Analysis of preferences by respondents from different medical institutions

As shown in Table 4, it was clear that *maintenance price* to primary hospitals matters more critically than *maintenance quality*, while quality matters more critically to tertiary hospitals. The different focus was related to the overall strength and performance of different medical institutions. Primary hospitals usually had less investment or resources assigned to medical device service. Hence, the maintenance quality was sacrificed as a trade-off to lower maintenance price. In contrast, tertiary hospitals’ overall performances and capabilities were stronger to afford more expensive maintenance to ensure high maintenance quality. Although there was a difference in ranking factor importance, primary and tertiary hospitals’ most desirable maintenance service profile was identical.

## Discussion

### ***The influences of****factors****on medical endoscope maintenance service***.

In evaluating maintenance service of medical endoscopes, medical staff, and engineers put a stronger emphasis on two factors, i.e., *maintenance quality* and *maintenance response*, and less attention was given to *service provider*, *maintenance price*, and *efficienc*y. Users of medical endoscopes, represented by medical staff and engineers in this research, paid more attention to the maintenance quality of medical endoscopes and were not willing to accept the endoscope failure during use. Medical endoscopes are essential and commonly used medical devices in the medical examination process; when a malfunction occurs, the users expect prompt responses from the service providers to resolve the problem and maintain continuity in examination and treatment.

Since public hospitals benefit from partial financial subsidies from governments, they can afford high-quality (with relatively higher prices) medical endoscope maintenance service. They were not tolerant of low-price and low-quality maintenance service, nor were they interested in costly service [34].

Although *service provider* is a less important factor, the hospitals were resistant to *third-party service providers*. This result is attributed to the poor standards and the unreliable quality level of the third-party service [35, 36]. *Maintenance time* is the slightest concern. Medical endoscopes require high maintenance quality, which takes a relatively long maintenance time as a medical device. It took a long maintenance time to repair or maintain endoscopes, and this was generally accepted, especially when the service providers could offer alternative endoscopes to use.

### Medical staff and engineers have different preferences for maintenance service

There was no significant difference between the medical staff and the medical engineer groups in ranking the five factors. Medical staff and medical engineers put a strong emphasis on maintenance response, quality, and price. Medical staff was lack of knowledge of the causes or mechanisms of endoscope failures; as endoscope users, they needed prompt responses from service providers, thereby enhancing their understanding of the impacts of the failure on the diagnosis/treatment and giving them psychological support. Therefore, medical staff assigned higher preference scores to *maintenance response*. However, as providers of daily maintenance of medical endoscopes, medical engineers better understood endoscopes’ operation mechanisms and working principles. When the medical endoscopes malfunctioned during use, they paid more attention to the causes of the malfunction, troubleshooting the problems, and proposing solutions to fix the problems and avoid them in the future. With different emphasis and expectations on the maintenance service provision, medical engineers also assigned higher scores to maintenance response and maintenance quality.

Furthermore, in the event of a failure of endoscopes, medical engineers did not experience the same level of nervousness as medical staff, so they did not rate *maintenance response* as high as the medical staff. On the contrary, as users of endoscopes, medical staff did not understand the technical requirements of endoscope maintenance, so they had a preference for the service provided by the original manufacturer. Compared with medical engineers, medical staff assigned a higher score (0.450) to the service provision by the original manufacturer.

### Primary hospitals and tertiary hospitals had different preferences on maintenance service

By analyzing the factor importance rated by respondents from different medical institutions, we found that *maintenance response* was the most critical factor for primary and tertiary hospitals. This result is due to the similar reasons discussed above, which led to medical staff and medical engineers assigning the highest score to this factor.

Regarding the second most crucial factor, tertiary hospitals emphasized *maintenance quality* while primary hospitals focused on *maintenance price*. It was related to the regional economic capacity, comprehensive strength of medical institutions, and operation mechanism of medical institutions. In recent years, private medical institutions in China have developed rapidly, and some hospitals have further expanded in groups and chains. This study observed that primary hospitals that rely on government funding were more cautious in terms of operating expenses [37]. This situation was reflected in the strong emphasis on *maintenance price*, higher than the score rated by tertiary public hospitals. As high-end medical device, medical endoscopes were widely used in higher-level medical institutions, but less used in low-level and private medical institutions was relatively low. Having a solid orientation towards low-price endoscope maintenance service puts the quality of maintenance at risk, leading to defective endoscopes used for diagnosis or examination. This situation is an important issue that is noteworthy and needs prompt action. According to the attributes of hospitals, hospitals can be divided into three different comparison groups: primary and tertiary hospitals, general and specialist hospitals, public and private hospitals. Primary hospitals and private hospitals are more sensitive to the price factor than tertiary and public hospitals. Considering the further opening of China’s medical market and the vigorous development of private institutions, it can be predicted that demands for high-end and low-end medical products and services will continue to co-exist in China’s medical market for a long time [38]. It is, therefore, imperative that medical product and service providers develop a comprehensive portfolio of products and services, to meet hospitals’ diverse needs and specifications for functions, features, services, and prices [39, 40].

## Selection and information bias

The experimental study design means that selection bias and information bias might exist, which is the limitation of this research. Selection bias could result from selecting the respondents (subjects) in the conjoint analysis, limiting the comparability between groups (medical staff and medical engineers; primary and tertiary hospitals) being studied. To reduce the impacts of selection bias, the sampling method used in choosing respondents was random, and the professionals who met the criteria had an equal probability of being included in the study. Future work will expand the conjoint analysis to include more subjects and refine the conjoint analysis results further.

A questionnaire helps collect perspectives, views, and opinions on the preferences of endoscope service factors. However, information bias may arise from self-reporting bias (recall bias) or inaccurate estimation. To overcome recall bias, we defined the selection criteria to choose respondents (subjects) to participate in the questionnaire, requiring more than 3 years of experience in using or managing medical endoscopes. Therefore, these respondents were supposed to have up-to-date knowledge to evaluate the service factors. To ensure the internal validity of the collected responses and minimize the impacts of inaccurate estimation, Pearson’s correlation coefficient and Kendall’s tau were calculated to check the reliability and validity of the regression model and estimated utility values.

The next phase of the study will involve surveys with a broader group of respondents who will rate the service factors. In addition to using statistical methods, we will compare the survey data and the results from conjoint analysis with users’ Evaluation reports or Technical reports on medical endoscopes to examine the validity and reliability of the self-reporting instrument.

## Conclusions

By employing conjoint analysis, we successfully identified the factors influencing the selection of maintenance service for medical endoscopes and their respective importance. Our analysis, conducted at three levels: aggregate, occupation, and medical institution, allowed for a comprehensive examination of service factors. This method facilitated the accurate determination of significant factors and their corresponding weights that impact the maintenance service of medical endoscopes. In our analysis, we considered diverse perspectives from healthcare professionals across different positions and the varied resources and capacities of medical institutions, ensuring a thorough assessment of the demand for medical endoscope maintenance services [41]. The insights gained from our analysis served as decision-making tools, leading to favorable outcomes in the procurement of endoscope maintenance services across 50 collaborating hospitals. This study presents compelling evidence endorsing the effectiveness of the conjoint method in evaluating the actual demand for maintenance services for medical endoscopes within the Chinese market.

This study makes a contribution by employing the conjoint analysis method to integrate and evaluate a comprehensive list of important factors, providing a novel decision-making tool of selection process in medical institutions in China, addressing the oversight of critical factors such as the tiers of hospital (including hospital functions, service capabilities, and the level of medical care provided), and the users’ occupations, beyond prioritizing price. These critical factors could be embedded in an innovative tool to facilitate decision making at medical institutions in choosing the suitable endoscope device and the associated maintenance service, catering to hospitals of different levels and users with diverse occupational characteristics [42, 43].

Moreover, medical device service providers can use such a decision-making tool to customize service combinations. The result is a quantified expectation of the maintenance services more likely to be embraced by hospitals of varying levels and users with distinct occupational characteristics [44, 45].

In conclusion, this research attempts to address gaps in existing research and practice by presenting a fresh perspective and methodology for those responsible for procuring medical equipment and its post-sale services. It empowers decision makers to make informed choices about service options. Simultaneously, it encourages medical device suppliers and service providers to improve their practices.

Despite the strengths of the Conjoint survey design and conduct, this study has several limitations. Firstly, the eligibility criteria for choosing participants and hospitals may limit the generalisability of the findings. In addition, due to the online survey methodology and the nature of the convenience sampling method, our sample consisted of those with convenient access and those who were willing to share opinions on the maintenance service of endoscopes. Future work is needed to include moving various hospitals and participants to achieve the demographic, geographic, and socioeconomic diversity representatives of the endoscope users in China.

Secondly, research on preferences is limited in that the assigned preference weights are specific to the defined factors and levels. In Conjoint analysis, it is possible that some essential factors were not included, which may lead to inaccurate utility scores, as utility scores depend on the set of factors and levels used to define a product or service.

Thirdly, this study only describes the user preference of medical endoscopes, and it does not identify the factors that affect preferences and the causal relationships between them. Future work is needed to investigate such relationships.

### Electronic supplementary material

Below is the link to the electronic supplementary material.

**Supplementary Material: Table S1:** Maintenance Service Preference Questionnaire of Medical Endoscope

## Data Availability

The datasets used during the current study are available from the corresponding author on reasonable request.

## List of Abbreviations

SPSS: Statistical Product and Service Solutions
