# Supplementary material for: Decision tool of medical endoscope maintenance service in Chinese hospitals: a conjoint analysis
Source: BMC Health Serv Res. 2023 Dec 15;23:1424. doi: 10.1186/s12913-023-10458-y (PMC10724992; doi:10.1186/s12913-023-10458-y)
Supplement: Supplementary file 1 — Supplementary Material: Table S1: Maintenance Service Preference Questionnaire of Medical Endoscope [file 12913_2023_10458_MOESM1_ESM.pdf]

Maintenance Service Preference Questionnaire of Medical Endoscope

Information of Participants

Hospital name:		Hospital level:		Department:	
Director of the Department:		Occupation / Title:		Mobile Phone/Email:	
Participant Name:		Occupation / Title:		The years for reletive work of medical endoscope	

Note 1: The manufacturer's maintenance service is divided into five factors: maintenance quality, maintenance price, maintenance response, maintenance efficiency and service provider, each factor with 2~3 level values.

Note 2: According to the actual situation of your hospital, which kind of after-sales service would you prefer to receive, and rate your preference for each group of after-sales service combination.

Note 3: Scoring is based on the preference combination of maintenance service level indicators. The scoring criteria are divided into five levels: very important / very good = 10; important / good = 8~9; general / general = 6~7; unimportant / relatively bad = 3~4; very unimportant / very bad =1~2.

Sample: This combination roughly meets my requirements for endoscope service, but there are still some defects, so preference score is 7 .

Order	Maintenance Quality	Maintenance Prie	Maintenance EfficiencyMaintenance Response	Maintenance Efficiency	Service Provision	Preference Score (1-10)
1	Same Fault≤6 months	Hard Endoscope≤5000、Soft Endoscope≤10000	Maintenance Response≤3 days	Maintenance Efficiency≤20 days	Service provided by the original manufacturers	
2	Same Fault≤6 months	Hard Endoscope≤5000、Soft Endoscope≤10000	Maintenance Response≤3 days	Maintenance Efficiency≤30 days	Service provided by third party service providers	

3	Same Fault≤6 months	Hard Endoscope≤5000、Soft Endoscope≤10000	Maintenance Response≤1 day	Maintenance Efficiency≤10 days	Service provided by third party service providers	
4	Same Fault≤6 months	Hard Endoscope≤5000、Soft Endoscope≤10000	Maintenance Response≤1 day	Maintenance Efficiency≤10 days	Service provided by the original manufacturers	
5	Same Fault≤6 months	Hard Endoscope≤20000、Soft Endoscope≤50000	Maintenance Response≤1 week	Maintenance Efficiency≤10 days	Service provided by third party service providers	
6	Same Fault≤6 months	Hard Endoscope≤20000、Soft Endoscope≤50000	Maintenance Response≤1 day	Maintenance Efficiency≤20 days	Service provided by third party service providers	
7	Same Fault≤6 months	Hard Endoscope≤10000、Soft Endoscope≤30000	Maintenance Response≤1 week	Maintenance Efficiency≤10 days	Service provided by the original manufacturers	
8	Same Fault≤6 months	Hard Endoscope≤10000、Soft Endoscope≤30000	Maintenance Response≤1 day	Maintenance Efficiency≤30 days	Service provided by the original manufacturers	
9	6 months<Same Fault≤12 months	Hard Endoscope≤5000、Soft Endoscope≤10000	Maintenance Response≤1 week	Maintenance Efficiency≤30 days	Service provided by third party service providers	
10	6 months<Same Fault≤12 months	Hard Endoscope≤5000、Soft Endoscope≤10000	Maintenance Response≤1 day	Maintenance Efficiency≤10 days	Service provided by the original manufacturers	
11	6 months<Same Fault≤12 months	Hard Endoscope≤20000、Soft Endoscope≤50000	Maintenance Response≤3 days	Maintenance Efficiency≤10 days	Service provided by the original manufacturers	
12	6 months<Same Fault≤12 months	Hard Endoscope≤10000、Soft Endoscope≤30000	Maintenance Response≤1 day	Maintenance Efficiency≤20 days	Service provided by third party service providers	

13	Same Fault＞12 months	Hard Endoscope≤5000、Soft Endoscope≤10000	Maintenance Response≤1 week	Maintenance Efficiency≤20 days	Service provided by the original manufacturers	
14	Same Fault＞12 months	Hard Endoscope≤5000、Soft Endoscope≤10000	Maintenance Response≤1 day	Maintenance Efficiency≤10 days	Service provided by third party service providers	
15	Same Fault＞12 months	Hard Endoscope≤20000、Soft Endoscope≤50000	Maintenance Response≤1 day	Maintenance Efficiency≤30 days	Service provided by the original manufacturers	
16	Same Fault＞12 months	Hard Endoscope≤10000、Soft Endoscope≤30000	Maintenance Response≤3 days	Maintenance Efficiency≤10 days	Service provided by third party service providers	
